# Supplementary figures and images for: Neural and endocranial anatomy of Triassic phytosaurian reptiles and convergence with fossil and modern crocodylians
Source: PeerJ. 2016 Jul 21;4:e2251. doi: 10.7717/peerj.2251 (PMC4963226; doi:10.7717/peerj.2251)

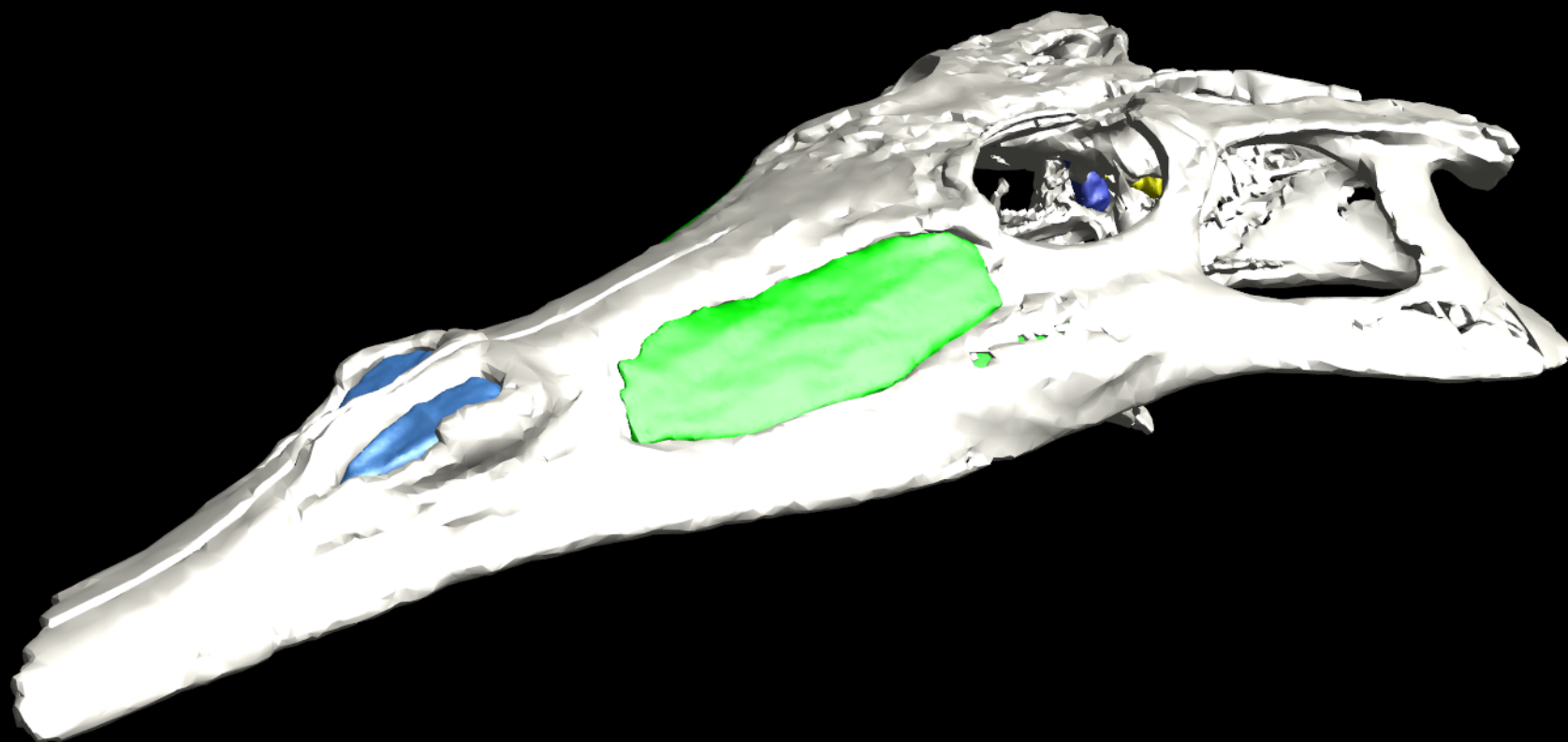

Supplement: Supplemental Information 1 [file peerj-04-2251-s001.pdf]

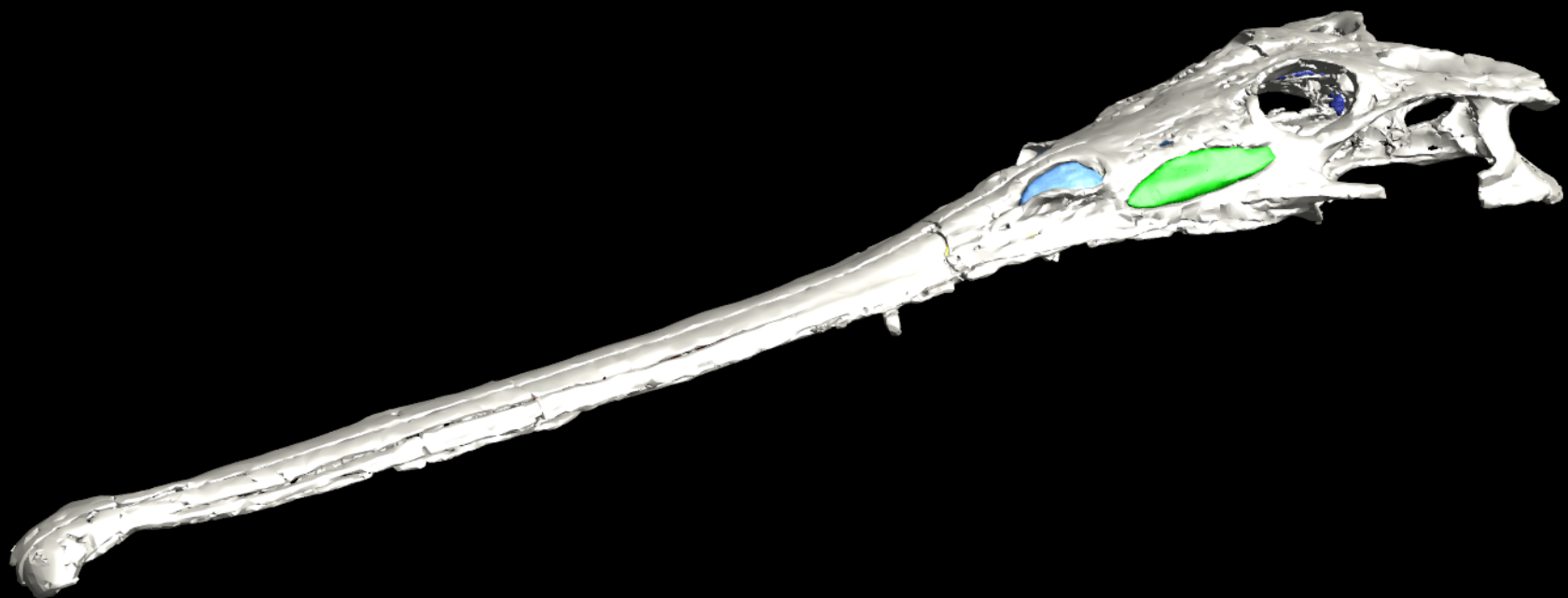

Supplement: Supplemental Information 2 [file peerj-04-2251-s002.pdf]
